# Clinical education: nursing students’ experiences with multisource feedback using a digital assessment instrument in the emergency medical Service - a qualitative study

**DOI:** 10.1186/s12909-025-06950-0

**Published:** 2025-03-18

**Authors:** Tomas Nilsson, I. Masiello, E. Broberger, V Lindström

**Affiliations:** 1 Department of Clinical Science and Education, Karolinska Institutet, Södersjukhuset, Stockholm, 11883 Sweden; 2 Department of Computer Science and Media Technology, Linnaeus University, Växjö, Sweden; 3 Department for Neurobiology, Care Sciences, and Society, Division of Nursing, Karolinska Institutet, Huddinge, Sweden; 4 Department of Nursing, affiliationision of Ambulance Service, Region Västerbotten, Umeå University, Umeå, Sweden

**Keywords:** Emergency services, Formative feedback, Multicourse feedback, Reflection, Clinical education, Nursing

## Abstract

**Background:**

Clinical education in Emergency services (EMS) is unique due to its dynamic environment, brief patient encounters, and unpredictable cases. EMS provides valuable learning opportunities for nursing students, fostering person-centered care approaches and a variation of clinical training and learning. Formative feedback is crucial to develop knowledge and skills. Multisource feedback (MSF) offers a comprehensive assessment by incorporating feedback from various individuals, promoting self-reflection and targeted learning. MSF has not, to our knowledge, been systematically evaluated in the context of EMS, and therefore, the aim of the study was to describe nursing students’ experiences with MSF during their clinical education in the EMS, using a digital instrument as a facilitating tool.

**Methods:**

A qualitative design with an inductive approach was used. Data were collected in 2021, using focus group interviews (*n* = 4) with 31 final-semester nursing students in Stockholm, Sweden, who had conducted clinical education in the EMS and received MSF through a digital instrument. Data were analyzed using reflexive thematic analysis, guided by Braun and Clarke’s methodology.

**Results:**

Three themes revealed: feedback from sources familiar with the student’s learning objectives, feedback from sources unfamiliar with the learning objectives, and general perceptions of MSF in the EMS. Students valued self-reflection and feedback from peers and supervisors for personal and professional growth. Patient feedback was challenging due to their limited contextual understanding and emotional states, while feedback from other healthcare professionals was appreciated but hindered by the healthcare professionals’ workload and timing constraints. Overall, students appreciated MSF’s diverse perspectives, enriching their learning, performance, and development.

**Conclusion:**

This study underscores the value of MSF in nursing students’ clinical education within the EMS. Feedback from peers, supervisors, and self-reflection enhances self-awareness, professional growth, and mutual support. Despite challenges like stress and logistical barriers, structured support and a digital instrument improved accessibility and alignment with learning objectives for the students. Incorporating patient and healthcare professionals’ feedback enriches education by promoting patient-centred care and collaboration. MSF supported reflective practice, and team dynamics and highlights the need for refined feedback processes to optimize learning and professional development for nursing students during clinical education.

**Supplementary Information:**

The online version contains supplementary material available at 10.1186/s12909-025-06950-0.

## Background

Clinical education in the Emergency Medical Service (EMS) is characterized by brief patient encounters, dynamic environments, and diverse patient complaints [1–3]. Unlike other healthcare settings, the EMS offers limited preparation for the students before the patient encounter since the cases cannot be predicted and predetermined [2, 4]. Despite these challenges, research suggests that students can learn professional nursing skills and about caring with a holistic approach within the EMS context. Through hands-on training and exposure to diverse patient encounter, students enhance their practical expertise and critical thinking [5, 6]. However, effective learning requires skilled supervisors to facilitate and support the learning process [7]. Globally, EMS staffing varies widely, ranging from emergency physicians with advanced university education to emergency medical technicians (EMTs) with non-university-level training, resulting in significant competency differences between EMS staff and teams [8]. In Sweden, EMS teams are staffed with at least one registered nurse (RN), commonly holding an additional year of training in emergency care. In Stockholm, the setting of this study, EMS teams include at least one RN with additional training, while the other team member may be an EMT or RN [9]. In the Swedish EMS, the traditional model of clinical supervision pairs students with a single primary supervisor, aiming to offer structure and support the student’s learning progression [5]. However, this supervision model risk becoming one-sided, as it relies solely on the supervisor’s observations and perspective, potentially introducing bias into the feedback and assessment [10, 11]. The bond between a single supervisor and student may also lead to subjective feedback and assessments [12, 13]. Organizational challenges, such as high workloads, shiftwork, and supervisor absences, further complicate the model of using single supervisors [14].

Commonly, during clinical education, supervisors use assessment instruments based on learning objectives (LOs) to support nursing students in acquiring essential skills, evaluate performance, identify strengths, and highlight areas for improvement. For these assessments to be effective and constructive, supervisors must thoroughly understand the assessment instrument and possess both clinical expertise and supervision experience [15–17]. Raustøl et al. conclude that assessments often rely more on supervisors’ subjective standards and intuition than on the provided assessment instruments or university guidelines [18]. During clinical education, nursing students usually undergo two structured assessments: a mid-point summative assessment, which facilitates discussions on strategies for improvement, and a final assessment focused on grading [19]. Unlike timely formative feedback, these summative assessments are disconnected from the clinical workflow and often provide feedback at a general level. Timely and regular feedback is a cornerstone of student learning in clinical education. Effective feedback provides critical insights into students’ progress, reinforcing good practices, fostering self-reflection, and motivating growth [17, 18]. To ensure its impact, feedback must be delivered promptly, follow clear criteria, and need to be integrated into clinical education as a natural daily routine [20]. Additionally, discussions between supervisors and students are vital for delivering high-quality feedback that facilitates meaningful learning [21–23]. Formative feedback provides continuous feedback on strengths and areas for improvement, enabling students to reflect and adjust care in real-time, thereby maybe minimizing feedback distortion [24]. The feedback is intended to be direct and actionable to foster a dialog between the supervisor and the student, rather than merely delivering instructions [25]. When effectively implemented, formative feedback supports a dynamic learning environment tailored to students’ needs [26].

Multisource feedback (MSF) in clinical nursing education uses feedback from peers, patients, supervisors, and healthcare professionals, offering a 360-degree assessment of a student’s skills, communication, professionalism, and teamwork [27–29]. MSF fosters self-reflection and dialogue by enabling comparisons between self-assessments and feedback from other sources to identify discrepancies and areas for improvement [24]. MSF in nursing education aim to enhances clinical skills through diverse feedback, providing broader learning insights while emphasizing the importance of patient perspectives in the learning environment [30, 31].

In the EMS, MSF can provide valuable formative feedback by integrating multiple perspectives, potentially reducing bias through diverse sources of input [32]. To organize and structure feedback obtained through MSF more effectively, digital assessment instruments can play a pivotal role. Previous studies have explored the use of digital instruments like e-portfolios, which facilitate the collection, documentation, and organization of feedback from diverse sources. These instruments provide students with accessible insights into their progress, serving as valuable resources for reflective discussions or grading purposes when aligned with the intended LOs [33, 34]. In this context, the digital instrument enables the integration of MSF creating a platform to gather insights from supervisors, peers, and patients to provide a broader view of a student’s competencies. These insights identified in prior research led to the aim of the study, which was to describe nursing students’ experiences with MSF during their clinical education in the EMS, using a digital instrument as a facilitating tool.

## Methods

### Study design

This study with a qualitative study design utilized focus groups, guided by a semi-structured interview guide. Focus groups were chosen to facilitate collective discussion on students’ experiences with MSF, enhancing recall, interaction, and dialogue and, encouraging diverse perspectives during the focus group interview [35]. The collected data was analyzed using reflexive thematic analysis outlined by Brown and Clarke [36]. The study was systematically organized following as outlined in COREQ (described in appendix 3). Codes, categories, and themes can be reviewed in Appendix 1 [37, 38].

### Multisource feedback using a digital instrument

During their clinical education, students utilized a digital instrument to collect MSF using the Ambulance Assessment Instrument (AAI) as the designated assessment instrument. The AAI is an adaptation of the standard assessment instrument named Assessment of Clinical Education (ACIEd) used in nursing education [19]. The adaption of the instrument was validated by Nilsson et al. [39] by using 50 supervisors who assessed students’ performance in four simulated patient encounters using both AAI and ACIEd. In the study, no significant differences in pass/fail gradings using the two assessment instruments were found. The AAI instrument contains 13 LO`s derived from the course curriculum. Four of the LO`s was especially aimed for patients and their next of kin. Two LO `s was specific for other healthcare professionals, for example, RNs at the emergency department. Seven LO`s were specific for supervisors, students, and peers. In addition, supervisors, students, and peers were intended to use all 13 LO`s. The AAI lacks clear assessment criteria due to the varying complexity of patient cases, requiring supervisors to rely on their expertise to evaluate students based on each case’s specific challenges. The AAI used a Likert scale ranging from one to seven, where one was described as “Not at all,” and seven was described as “To a very high degree.” The AAI can be found in Appendix 2.

To collect feedback from the different available sources, the students used the time between care encounters. The time available to collect the feedback varied from minutes to hours, depending on the daily workload in the EMS. Students received weekly summaries of their assessments during Monday gatherings led by a clinical supervisor, who coordinated and supported them throughout their clinical education. Since the software used in the study lacked an easy way to extract historical data, the first author and supporting clinical supervisors prepared these summaries. The summaries were individually presented as pie charts and discussed at a general level. Individual discussions with clinical supervisors were also offered to address individual questions about the assessments.

### Setting

This study was conducted in 2021 in the EMS company owned by the county “Ambulansen I StorStockholm AB (AISAB)” in Stockholm, Sweden. The students were placed at two ambulance stations located in densely populated areas with a high caseload. In accordance with Wallin et al. (2013) high caseloads in the EMS are essential for a rich learning environment due to the variety of patient encounters [5]. The clinical education was conducted at all hours of the day, totaling 32 h per week over the six-week period.

The EMS supervisors were RNs with an additional year of training in emergency care and at least one year of employment within the EMS. During students’ clinical education, supervisors were responsible for providing pedagogical guidance through feedback following the course curriculum outlines in the LO`s. All supervisors had pedagogical education in their basic nursing education, and most supervisors have conducted a shorter pedagogical education provided by the region. Pedagogical support for the clinical supervisors could be found in the EMS organization by a supervisor specifically tasked with coordinating and supporting clinical education in the service. EMTs were a part of the EMS team and participated in the daily pedagogical work.

### Participants

A convenience sample of nursing students was utilized for this study, selected based on their availability to participate during their clinical education in the EMS [40]. The participants were recruited from a university nursing program in Stockholm, which spans six semesters over three years and awards 180 credits within the European Credit Transfer System, culminating in a bachelor’s degree. The study recruited students in their final semester, where the curriculum focused on emergency care. All 32 students participating in clinical education during the study period were invited to participate, and 31 agreed to participate. The non-participating student declined due to concerns about potential negative impacts on his/her studies. Comprehensive written and oral information about the study and a consent form were provided to all participants before the students started their clinical education.

### Data collection

Four focus groups were conducted after completing students’ clinical education in the EMS, and all grades were finalized. Three focus groups consisted of eight students, and one consisted of seven students. The focus groups were facilitated by the first author and assisted by one clinical supervisor who coordinated and supported the students during their clinical education. As the first author was well known by the participants and took part in the data collection, subjectivity was unavoidable and considered an advantage in generating high-quality data following Brown and Clark [36]. The researcher used the interaction to keep the discussion on topic without hindering the debate between the participants while the clinical supervisor took notes [35]. The focus group flowed naturally with an encouraging interaction from the researcher as stated by the methodology [41].

A semi-structured interview guide was designed for this study to guide the discussions in the focus groups. The guide was divided into three sections:

1. **Introduction** included a brief explanation of the study’s aim, a confirmation of voluntary participation, and an assurance that the discussion during the focus groups would be audio-recorded.
2. **Body** focused on the students’ experiences with MSF, beginning with an open-ended question, “Can you describe your experience of multisource feedback?” Follow-up probing questions were asked to delve deeper into the discussions. The probing questions were primarily designed in short sentences, for example, “Could you elaborate on that statement?”. On several occasions, probing questions were used to engage the rest of the group and were designed as follows: “What is the rest of the groups’ opinion on that matter.”
3. **Summary** provided an opportunity for participants to discuss any additional topics not previously covered.

Each focus group lasted between 45 and 70 min. The interviews were recorded using a voice recorder on two separate phones in flight mode, one main phone and one as backup, recordings were transcribed verbatim.

### Data analysis

Reflexive thematic analysis was employed to analyze the four focus groups. The method was chosen due to the nature of the data and the study’s aim. To enhance methodological coherence, this study adhered to the ten recommendations proposed by Braun and Clarke [34]. A non-positivist “Big Q” qualitative perspective was adopted, emphasizing subjectivity and recognizing the researcher as an active participant in the data generation, using contextual knowledge to understand the data [35]. Within this framework, bias is intentionally acknowledged and leveraged as a resource to deepen understanding of the research context and the nature of participants’ experiences. The study aim directed the coding process, ensuring that the construction of themes was in line with the study’s aim. Initially, the recordings from the focus groups were transcribed and reviewed multiple times to enable the researcher to become deeply familiar with the data. In the subsequent phase, initial condensed units were identified. These units were further condensed into codes, which were then examined for patterns and similarities. Related codes were grouped together to reduce data complexity and identify broader trends within the dataset. Finally, these grouped codes were organized into categories. Following this, themes were carefully constructed with the intent of being a meaning-based interpreted story [37]. Lastly, the themes and categories were linked back to the narrative, ensuring that the themes accurately represented the complete story and that each theme was distinct and informative. Finally, the report was compiled using the identified themes and categories. The research team was closely involved in constructing of categories and the themes and how they fit with the narrative.

### Rigour

To ensure trustworthiness in this qualitative study, the principles of credibility, dependability, and transferability need to be addressed. Credibility in this study was ensured by including 31 nursing students in their final semester, who were assigned for clinical education in the EMS without special selection, making them representative of the broader cohort of nursing student. The data collection reflected the EMS context, and the analysis process was reinforced through research team, which holds extensive experience from the context, the nursing education as well as methodological experience. Discussions, ensuring rigorous data interpretation was preformed [42]. Dependability was ensured by standardizing the interview process. All interviews were conducted by the first author with the support of a clinical supervisor, using a semi-structured interview guide. This approach ensured that all participants were asked the same initial questions while allowing follow-up questions to vary based on individual responses. To enhance transferability, the study provides a description of the research context, participant’s academic background, data collection methods, analytical procedures, and presentation of findings. This transparency allows readers to assess the applicability of the study’s findings to other contexts [43].

## Results

The themes, categories and codes were identified through reflexive thematic analysis, supported by representative quotations from students, capturing their diverse perspectives on MSF when utilizing a digital assessment instrument. Three major themes emerged from the analysis of data collected from four student focus groups, with each theme encompassing various categories that reflect the nuanced experiences and insights of the participants.

Each theme is described in detail through its corresponding categories, which provide a deeper understanding of the student’s experiences with the feedback process. These themes, along with their categories, offer a comprehensive overview of how students perceive MSF, highlighting its positive impacts as well as areas requiring improvement. Table 1 presents a summary of the themes and categories that emerged from the qualitative analysis, along with illustrative quotations from both students and faculty/administrators, providing further context. Below, each theme is described in greater detail to enhance the understanding of the findings.

**Table 1:** Table 1

Themes	Categories	Sample quotations
Feedback from sources familiar with the LOs*	Self-reflection Feedback from peers Feedback from supervisor	*Then I recalled: What did I do regarding the LO? Did I do it well*,* and what could I have done differently.* *I am so critical towards myself that it becomes straining.* *I could support my peer using the peer-assessments.* *I feel that if I don’t get feedback then I become lost*
Feedback from sources unfamiliar with the LOs	Feedback from patients Feedback from other care professionals	*The patients are not familiar with what we students do.* *I could not accept the feedback provided. I knew I did nothing wrong.* *The handover needs to be comprehensible for the one who receives it and is supposed to pass it on in the organization wherefore*,* his or her feedback is super important.* *Everyone is sitting inline*,* waiting to give their handover rapport and it doesn’t feel like the right time*
General perceptions of MSF** in the EMS***	Context Multisource feedback in the EMS	*Finding the right time for feedback is a challenge.* *It would be easier*,* definitely. In a nursing department.* *This instrument would be amazing to show at the assessment conference with all the different assessments. We could say*,* “Look*,* this is how the student has developed and progressed*,* with all these measurements and assessments.”*

* LO- Learning Objectives ** MSF- Multisource Feedback *** EMS- Emergency medical service

### Feedback from sources familiar with the learning outcomes

Students discussed diverse experiences related to feedback from sources familiar with the LOs. These experiences were organized into categories based on the sources of the feedback, resulting in the following three categories: Self-reflection, Feedback from peers, and Feedback from supervisor.

Students generally described the experience of self-reflection as positive. This feedback instigated a personal reflection on the student’s performances. The insights that the students had then facilitated discussions with their supervisors and their peers, leading to constructive feedback from both parties.

*“Then I recalled: What did I do regarding the LO? Did I do it well*,* and what could I have done differently?” (Focus Group 1)*.

*“A bit of self-reflection is good.” (Focus Group 1)*.

The students described being more critical in their reflections on their own performances compared to other sources like supervisors or peers. Therefore, the discussions with peers and supervisors were crucial to transform self-criticism into constructive feedback. Two student’s comments illustrated this:

*“You don’t want to give yourself a seven (using the 1–7 Likert scale)*,* you can always do better.” (Focus Group 4)*.

*“I am so critical towards myself that it becomes straining.” (Focus Group 1)*.

Overall, the students concluded that self-reflection, combined with supervisory feedback, was beneficial for their learning since it motivated them to reflect on their performances instead of simply performing tasks. The students also concluded that more frequent self-reflection would be beneficial. They suggested that self-reflection could enhance the quality of care they provide and their ongoing development toward becoming RN. This was exemplified by one student saying:

*“I believe that if I would have self-reflected more regularly*,* like every day*,* at least once a day would have been fun.” (Focus Group 2)*.

Students described feedback from peers as a positive experience. They noted that such feedback facilitated discussions and encouraged peer dialogue, thereby supporting each other’s learning and development towards becoming RN. Reflecting on their experiences with peer feedback during caregiving encounters, students remarked:

*“Feedback from peers was educational.” (Focus Group 1)*.

*“Feedback from peers created discussions.” (Focus Group 4)*.

*“Reflections instigated dialog.” (Focus Group 4)*.

The students highlighted that feedback from peers fostered a supportive environment, enabling them to assist one another. They discussed that feedback from peers encouraged reflection and cultivated a sense of camaraderie and mutual support. This support was particularly valuable given the recurring sense of vulnerability students often experience during their clinical education. The students said:

*“We saw each other’s progression.” (Focus Group 1)*.

*“I could support my peer using the peer-assessments.” (Focus Group 2)*.

However, the students also discussed the potential challenges of providing feedback to peers who were struggling with their learning progress. This related to the fact that the students worked closely together during their clinical education and criticism could jeopardize the comradery and the team spirit. Furthermore, the students identified that different personal goals during clinical education could be hampering and could provide challenges in giving feedback. The students exemplified this by saying:

*“It would probably be hard to provide feedback if someone was performing badly.” (Focus Group 3)*.

*“It would be difficult if the level of ambition differed.” (Focus Group 4)*.

On the other hand, students discussed the supporting nature of the assessment instrument. Students described that the assessment instrument could be helpful in situations where constructive criticism could be challenging to convey. The assessment instrument provided structure and a sense of objectivity for the peer. The assessment instrument helped make the feedback less dramatic for the receiving party. A student said:

*“Couldn’t it be good and nice if you could use the assessment instrument as a tool for giving feedback? Then you don’t need to confront your peer.” (Focus Group 3)*.

Students regarded feedback from supervisors as essential for their professional development. The feedback provided critical direction, without which they felt disoriented. Additionally, the feedback contributed to students feeling recognized as individuals. The students said:

*“You want to know that you’re on the right path.” (Focus Group 4)*.

*“I feel that if I don’t get feedback then I become lost.” (Focus Group 4)*.

*“Feedback is very important for me to feel seen.” (Focus Group 4)*.

The students also discussed the potential benefits of anonymity in supervisor feedback, suggesting it might lead to less biased feedback. Students discussed that the supervisors might avoid giving negative feedback to avoid confrontation which is exemplified by one student statement:

*”Feedback could be done anonymously*,* then it becomes a fairer assessment.” (Focus Group 2)*.

Moreover, students expressed a desire for more constructive feedback from their supervisors. The feedback from supervisors was seen as a cornerstone of student education and, thereby, as the most important.

*“I would have wished for more*,* more constructive feedback.*” *(Focus Group 1)*.

### Feedback from sources unfamiliar with the learning outcomes

Students’ experiences of feedback from sources unfamiliar with the LOs were diverse in nature. These experiences were organized into categories based on the sources of the feedback, resulting in the following two categories: Feedback from patients and Feedback from other care professionals.

Students described difficulties in gathering feedback from patients. Various issues were highlighted, particularly the patients’ inability to provide informed feedback on LOs they had no prior knowledge about. Additionally, patients limited understanding of the educational process made their feedback seem arbitrary. As some students remarked:

*“Patients and next of kin are so unknowing of the meaning of the clinical education. The feedback becomes unfunded.” (Focus Group 1)*.

*“We had a discussion with a patient*,* and he didn’t even understand the meaning of the words.” (Focus Group 2)*.

*”The patients are not familiar with what we students do.” (Focus Group 1)*.

The students also claimed that patients’ medical and psychological status affected their ability to provide feedback. The students reflected on the fact that they felt that patients tended to focus solely on themselves in their time of need thereby not being able to provide feedback on staff performances. They also claimed complications related to the patient’s age were elderly patients and children struggled with the quantity of the LOs. The students stated:

*“It is hard to ask for assessments when someone is filled with anxiety and only wants to be helped.” (Focus Group 2)*.

Elderly and children primarily. It might be enough with five questions for them.

*(Focus Group 2)*.

The students also described feeling uncomfortable asking patients for feedback. The students also reflected upon prior statements about believing in patients’ medical and psychological status and reasoned that their prejudices might be the real obstacle in asking for feedback.

*“For me*,* it felt hard if it concerned patients and next of kin.” (Asking for feedback)*.

*(Focus Group 1)*.

*“I believe that it is more about me feeling uncomfortable with handing it over to the patient (the assessment instrument). It is more about me than the patient or the instrument.” (Focus Group 1)*.

Some students also had negative feelings about feedback provided by patients. These concerns were related both to how the feedback was perceived and to potential biases in the feedback. Students discussed that they struggled with accepting negative feedback from patients which was not in line with their reflections on their performances. Students noted that they tended to ask for feedback from patients with a more positive demeanor, which often resulted in receiving more praise than constructive criticism.

*“I could not take to me the feedback provided. I knew I did nothing wrong.” (Focus Group 3)*

*”It naturally happens that patients that respond perhaps are those who are more satisfied patients from the start*,* so it becomes skewed.” (Focus Group 4)*.

*“As I said*,* a certain patient group is selected when asking for assessments.” (Focus Group 3)*.

The students also described positive experiences with feedback from patients. The students recognized that the patient’s perspective of the care provided, and that information could be beneficial for their development. The students also recognized that their own and the supervisor’s reflections on the care provided could dilute the information and skew the perspective.

*”It is very valuable to know how you handled the situation.” (Focus Group 1)*.

*“It is good to get insights on the patient perspective.” (Focus Group 3)*

*“They can provide more details about how they have experienced the encounter instead of the supervisor telling me how the caring encounter was.” (Focus Group 4)*.

The students expressed generally positive views about other care professional’s feedback. They emphasized the value of receiving insights from diverse perspectives within the healthcare setting, noting the particular importance of feedback from RNs who directly receive handovers. The students also discussed expanding feedback from other care professionals to additional sources due to its invaluable perspective on their performance. The students articulated that:

*“The handover needs to be comprehensible for the one who receives it and is supposed to pass it on in the organization wherefore*,* his or her feedback is super important.” (Focus Group 1)*.

*“It feels superb to get feedback on the handover” (from the receiving RN) (Focus Group 2)*.

*“Additional sources involved in patient care would have been beneficial.” (Focus Group 1)*.

However, students also identified challenges in receiving feedback from other healthcare professionals, mainly due to workload and timing constraints. The students discussed whether the feedback from a stressed healthcare professional could be overly negative due to the negative attitude of the student in question. They also discussed that the feedback could be given in a sloppy matter due to the workload and the unwillingness to give feedback. They highlighted several issues related to this.

*“There was no time for that.” (Focus Group 1)*.

*“They are already signing because it takes longer for us.” (Focus Group 2)*

*“Everyone is sitting inline*,* waiting to give their handover rapport and it doesn’t feel like the right time.” (Focus Group 4)*.

### General perceptions of multisource feedback in emergency medical service

The students also discussed the contextual influence of MSF and MSF in general in relation to their learning progress. This resulted in two categories: Context and Multisource feedback in the EMS.

During the focus groups, students highlighted several contextual challenges they faced regarding MSF during their clinical placements, comparing their experiences across different clinical environments. They noted specific obstacles in the EMS with the high stress setting and patients with medical and nursing needs that those students seldom had encountered previously in their education. The inherently stressful nature of working in ambulances and the difficulty in finding appropriate times to provide feedback were significant issues. The students said:

*“It is very stressful in the ambulance.” (Focus Group 4)*.

*“Finding the right time for feedback is a challenge.” (Focus Group 2)*.

*“It becomes a discussion for like 15 minutes or so for every patient and that kind of time doesn’t exist.” (Focus Group 1)*.

The students suggested that wards within the hospital might be more conducive to MSF. They emphasized the advantages of longer patient stays, which facilitate the development of relationships between students and patients. This setting also allows the possibility of leaving the assessment instruments with patients and returning later, giving patients more time to provide helpful feedback. The students commented:

*“It would be easier*,* definitely.” (In a nursing department) (Focus groups 2 & 3)*.

*“You see the patient more frequently over a longer period of time.” (Focus Group 4)*.

*“You can leave it and come back at a later time.” (The assessment instrument) (Focus group 1)*.

When discussing the pedagogical method, students generally regarded MSF positively. They appreciated the diverse perspectives, noting that it enriched the quality of team discussions and interactions with their supervisor. Students recognized that MSF offered more comprehensive and fair feedback, as it incorporated documented assessments from multiple sources. This multifaceted feedback provided a well-rounded view of their performance, which was valuable both for constructive discussions and for the grading process.

*“Because we had different supervisors*,* it felt like it gave a fair picture.” (Focus Group 2)*

*“This instrument would be amazing to show at the assessment conference with all the different assessments. We could say*,* “Look*,* this is how the student has developed and progressed*,* with all these measurements and assessments.” (Focus Group 4)*.

*“Many people have been involved in this*,* and the evaluations are interconnected.” (Focus Group 4)*.

## Discussion

The aim of the study was to describe nursing students’ experiences with MSF during their clinical education in the EMS, using a digital instrument as a facilitating tool. The main findings showed that self-reflection supported the students to identify strengths and areas for improvement, but without structure, it could lead to excessive self-criticism. As previous research shows, supervisors should guide students to maintain a balanced perspective, fostering actionable insights and reducing self-criticism, thereby enhancing the effectiveness of self-reflection in clinical education [44]. To address this, integrating structured self-reflection such as MSF, guided journals, or digital instruments, could provide a more constructive framework. Students highly valued peer feedback in MSF for fostering dialogue, support, and learning, particularly during vulnerable moments in clinical education. It enhanced teamwork, eased stress, and facilitated learning, though giving negative feedback was stressful, as noted in previous research [45]. To ensure feedback remains constructive, structured support is essential, therefore MSF assessment instrument should provide necessary guidance and thereby reduce emotional strain. Using a peer feedback construct outlining agreed-upon rules could mitigate the discomfort associated with delivering criticism, which is known to be difficult from prior studies [43]. Supervisors’ feedback was vital for MSF success, but also required supervisors to expand their roles beyond their traditional duties as supervisors, as feedback was provided from multiple sources. Effective MSF necessitates support through training on methodology and assessment instruments. Strengthening supervisor roles can involve collaborative strategies that promote shared learning and professional development, improving feedback delivery and optimizing the implementation of MSF [46, 47]. The results indicate that anonymous feedback mechanisms could promote fairness and honesty which could be facilitated by using the digital instrument.

As in a previous study, our findings showed that patient feedback posed challenges due to selection bias [48]. This highlights the need for structured approaches to engage a diverse range of patients, incorporating perspectives on care needs and individual differences. Integrating patient voices and guidance on feedback can promote patient-centred care and enhance learning. Feedback from healthcare professionals was highly valued by students but often restricted due to high-pressure environments and stressful handovers. To streamline the process, a dedicated digital platform, managed by the responsible nurse, could be pre-programmed with the selected, relevant LO`s, which can then be easily linked to the current student. This would allow students to review and discuss insights with supervisors and peers later, enhancing learning and reflection [49]. Nursing students acknowledged the inherently stressful nature of the EMS context which is earlier described by Nilsson et al. [47], emphasizing the need for support systems and dedicated forums where they can process their emotions and experiences. To address logistical challenges, shorter, focused feedback sessions could reduce pressure on students and supervisors [50]. Despite challenges of using the digital instrument and collecting feedback, students generally appreciated the pedagogical value of MSF, recognizing its ability to provide diverse perspectives and the fairness of feedback from multiple sources. The MSF offers a unique opportunity to not only foster learning but also create reflective, supportive moments for students. Through these discussions, students can explore their progression and gain emotional support [51].

### Limitations

A limitation of this study is related to the context of EMS. While EMS for clinical education requires rapid assessments and care for diverse patient complaints and needs, introducing MSF may have added stress for students, potentially influencing their perceptions of MSF. In less stressful clinical settings, where students have more time and a quiet space for assessment and feedback, the instrument might yield different results and experiences.

Variations in EMS systems, such as patient demographics, clinical protocols, and educational frameworks, pose challenges for comparing the effect and feasibility of MSF, as different systems may yield varying outcomes due to different demands. These disparities underscore the need for context-specific adaptations when applying our findings to different EMS systems. The transcripts were not sent to students for confirmation; however, they were given the opportunity to review them before analysis began. No student expressed interest in doing so. They were also informed that they could request access to their transcripts at any time if they changed their mind. This study was conducted after the COVID-19 pandemic, which introduced challenges in collecting MSF, particularly restrictions on students’ interactions with patients’ next of kin due to EMS protocols. These limitations likely impacted the breadth of students’ clinical experiences, as they had fewer opportunities to engage in person-centered care, including communication with next of kin. This restriction may also have altered both the learning environment and the way students applied the assessment instrument.

The interview guide was not piloted due to scheduling constraints, as student availability is limited to once per semester, and a pilot would have delayed data collection and the ongoing PhD project. Given that this study is the final in a series using similar methods, including interview guides, the research team was confident in its effectiveness for ensuring high-quality data.

## Conclusion

This study emphasizes the valuable role of MSF in nursing students’ clinical education within the EMS context, highlighting the impact of feedback from various sources on learning. Structured self-reflection enhances self-awareness and professional growth, while peer and supervisor feedback foster mutual support, though challenges such as stress were noted. The need for structured support to maintain constructive feedback, especially in peer and supervisor interactions, was identified. Despite barriers, integrating a digital instrument offers potential for more accessible, timely feedback aligned with learning outcomes. Overall, MSF encourages reflective practice, emotional support, and team dynamics, contributing to students’ professional development and calling for continued refinement of feedback processes. Further research focusing on improving the process of feedback from sources unfamiliar with the LO`s and the assessment instrument is needed.

## Electronic supplementary material

Below is the link to the electronic supplementary material.

Supplementary Material 1

Supplementary Material 2

Supplementary Material 3

## Data Availability

The dataset generated and analysed in this study is available from the corresponding author upon reasonable request.

## Declarations

A written, informed consent was sign by all participants before participation of the study. The study was designed according to the ethical principles for research described by the International Council of Nurses [40]. The study was approved by the Swedish Ethical Review Authority, Stockholm, Sweden (2016/594 − 31). All methods were performed in accordance with the relevant guidelines and regulations stipulated in the Helsinki declaration.

## References

[1] Nilsson T, Lindström V. Clinical decision-making described by Swedish prehospital emergency care nurse students - An exploratory study. Int Emerg Nurs. 2016;27:46–50. 26615949 10.1016/j.ienj.2015.10.006

[2] Sundström BW, Dahlberg K. Being prepared for the unprepared: a phenomenology field study of Swedish prehospital care. J Emerg Nurs. 2012;38(6):571–7. 22088772 10.1016/j.jen.2011.09.003

[3] Wallin K, Hörberg U, Harstäde CW, Elmqvist C, Bremer A. Preceptors´ experiences of student supervision in the emergency medical services: A qualitative interview study. Nurse Educ Today. 2020;84:104223. 31726285 10.1016/j.nedt.2019.104223

[4] Venesoja A, Lindström V, Castrén M, Tella S. Prehospital nursing students’ experiences of patient safety culture in emergency medical services-A qualitative study. J Clin Nurs. 2023;32(5–6):847–58. 35672936 10.1111/jocn.16396 PMC10083998

[5] Wallin K, Fridlund B, Thorén AB. Prehospital emergency nursing students’ experiences of learning during prehospital clinical placements. Int Emerg Nurs. 2013;21(3):197–203. 23140791 10.1016/j.ienj.2012.09.003

[6] Nilsson T, Lindström V. Nursing students’ perceptions of learning nursing skills in the ambulance service. Nurse Educ Pract. 2017;24:1–5. 28278433 10.1016/j.nepr.2017.02.011

[7] Conte H, Wihlborg J, Lindström V. Developing new possibilities for interprofessional learning- students’ experience of learning together in the ambulance service. BMC Med Educ. 2022;22(1):192. 35307011 10.1186/s12909-022-03251-8 PMC8935834

[8] Cooper S Jr., Grant J. New and emerging roles in out of hospital emergency care: a review of the international literature. Int Emerg Nurs. 2009;17(2):90–8. 19341994 10.1016/j.ienj.2008.11.004

[9] Hörberg A, Lindström V, Scheja M, Conte H, Kalén S. Challenging encounters as experienced by registered nurses new to the emergency medical service: explored by using the theory of communities of practice. Adv Health Sci Educ Theory Pract. 2019;24(2):233–49. 30443693 10.1007/s10459-018-9862-x PMC6483944

[10] Lockyer J, Sargeant J. Multisource feedback: an overview of its use and application as a formative assessment. Can Med Educ J. 2022;13(4):30–5. 36091727 10.36834/cmej.73775 PMC9441111

[11] Ten Cate O, Sargeant J. Multisource feedback for residents: how high must the stakes be? J Grad Med Educ. 2011;3(4):453–5. 23205189 10.4300/JGME-D-11-00220.1 PMC3244305

[12] Williams RG, Klamen DA, McGaghie WC. Cognitive, social and environmental sources of bias in clinical performance ratings. Teach Learn Med. 2003;15(4):270–92. 14612262 10.1207/S15328015TLM1504_11

[13] Johnson R, Browning K, DeClerk L. Strategies to reduce Bias and racism in nursing precepted clinical experiences. J Nurs Educ. 2021;60(12):697–702. 34870500 10.3928/01484834-20211103-01

[14] Axelsson C, Herrera MJ, Bång A. How the context of ambulance care influences learning to become a specialist ambulance nurse a Swedish perspective. Nurse Educ Today. 2016;37:8–14. 26596850 10.1016/j.nedt.2015.10.029

[15] Calcagni L, Lindell D, Weaver A, Jackson M. Clinical judgment development and assessment in clinical nursing education. Nurse Educ. 2023;48(4):175–81. 36728083 10.1097/NNE.0000000000001357

[16] Hernon O, McSharry E, MacLaren I, Carr PJ. The use of educational technology in teaching and assessing clinical psychomotor skills in nursing and midwifery education: A state-of-the-art literature review. J Prof Nurs. 2023;45:35–50. 36889892 10.1016/j.profnurs.2023.01.005

[17] Van de Ridder JM, Stokking KM, McGaghie WC, ten Cate OT. What is feedback in clinical education? Med Educ. 2008;42(2):189–97. 18230092 10.1111/j.1365-2923.2007.02973.x

[18] Natterøy CS, Tveit B, Hunskår I, Raustøl A. Suitable, fit, competent and safe to practice nursing? Assessing nursing students’ personal qualities in clinical placement-An integrative review. J Clin Nurs. 2023;32(17–18):6101–19. 37149742 10.1111/jocn.16747

[19] Ulfvarson J, Oxelmark L. Developing an assessment tool for intended learning outcomes in clinical practice for nursing students. Nurse Educ Today. 2012;32(6):703–8. 22051102 10.1016/j.nedt.2011.09.010

[20] Cantillon P, Sargeant J. Giving feedback in clinical settings. BMJ. 2008;337:a1961. 19001006 10.1136/bmj.a1961

[21] Branch WT Jr., Paranjape A. Feedback and reflection: teaching methods for clinical settings. Acad Med. 2002;77(12 Pt 1):1185–8. 12480619 10.1097/00001888-200212000-00005

[22] Allen L, Molloy E. The influence of a preceptor-student ‘daily feedback tool’ on clinical feedback practices in nursing education: A qualitative study. Nurse Educ Today. 2017;49:57–62. 27888784 10.1016/j.nedt.2016.11.009

[23] Duers LE, Brown N. An exploration of student nurses’ experiences of formative assessment. Nurse Educ Today. 2009;29(6):654–9. 19285761 10.1016/j.nedt.2009.02.007

[24] Kokkiz R, Inangil D, Turkoglu I. The effect of formative assessment on students’ clinical knowledge, skills and self-efficacy levels. Nurse Educ Pract. 2024;80:104120. 39213838 10.1016/j.nepr.2024.104120

[25] Koh LC. Refocusing formative feedback to enhance learning in pre-registration nurse education. Nurse Educ Pract. 2008;8(4):223–30. 17959416 10.1016/j.nepr.2007.08.002

[26] Wiles LL. Why can’t I pass these exams? Providing individualized feedback for nursing students. J Nurs Educ. 2015;54(3 Suppl):S55–8. 25688544 10.3928/01484834-20150218-02

[27] Violato C, Lockyer JM, Fidler H. Assessment of psychiatrists in practice through multisource feedback. Can J Psychiatry. 2008;53(8):525–33. 18801214 10.1177/070674370805300807

[28] Stenberg M, Mangrio E, Bengtsson M, Carlson E. Formative peer assessment in higher healthcare education programmes: a scoping review. BMJ Open. 2021;11(2):e045345. 10.1136/bmjopen-2020-045345 PMC7875268 33563627

[29] Donnon T, Al Ansari A, Al Alawi S, Violato C. The reliability, validity, and feasibility of multisource feedback physician assessment: a systematic review. Acad Med. 2014;89(3):511–6. 24448051 10.1097/ACM.0000000000000147

[30] Barr J, Ogden K, Robertson I, Martin J. Exploring how differently patients and clinical tutors see the same consultation: Building evidence for inclusion of real patient feedback in medical education. BMC Med Educ. 2021;21(1):246. 33926426 10.1186/s12909-021-02654-3 PMC8082899

[31] Caretta-Weyer HA, Kraut AS, Kornegay JG, Yarris LM. The view from over here: A framework for Multi-Source feedback. J Grad Med Educ. 2017;9(3):367–8. 28638519 10.4300/JGME-D-17-00200.1 PMC5476390

[32] Lui JK, Duke JD, Russo JV, Kelm DJ, Hinkle LJ. Multisource feedback in health professions education. Clin Teach. 2023;20(1):e13555. 36446412 10.1111/tct.13555

[33] Roa Romero Y, Tame H, Holzhausen Y, Petzold M, Wyszynski JV, Peters H, et al. Design and usability testing of an in-house developed performance feedback tool for medical students. BMC Med Educ. 2021;21(1):354. 34162382 10.1186/s12909-021-02788-4 PMC8220763

[34] Luther R, Richardson L. A web-based peer feedback tool for physical examination. Clin Teach. 2018;15(2):132–5. 28485102 10.1111/tct.12650

[35] Doody O, Slevin E, Taggart L. Focus group interviews in nursing research: part 1. Br J Nurs. 2013;22(1):16–9. 23299206 10.12968/bjon.2013.22.1.16

[36] Braun V, Clarke V. A critical review of the reporting of reflexive thematic analysis in health promotion international. Health Promot Int. 2024;39(3). 10.1093/heapro/daae049 PMC11132294 38805676

[37] Braun V, Clarke V. Toward good practice in thematic analysis: avoiding common problems and be(com)ing a knowing researcher. Int J Transgend Health. 2023;24(1):1–6. 36713144 10.1080/26895269.2022.2129597 PMC9879167

[38] Tong A, Sainsbury P, Craig J. Consolidated criteria for reporting qualitative research (COREQ): a 32-item checklist for interviews and focus groups. Int J Qual Health Care. 2007;19(6):349–57. 17872937 10.1093/intqhc/mzm042

[39] Tomas N, Italo M, Eva B, Veronica L. Assessment during clinical education among nursing students using two different assessment instruments. BMC Med Educ. 2024;24(1):852. 39112978 10.1186/s12909-024-05771-x PMC11308620

[40] Andrade C. The inconvenient truth about convenience and purposive samples. Indian J Psychol Med. 2021;43(1):86–8. 34349313 10.1177/0253717620977000 PMC8295573

[41] Wong LP. Focus group discussion: a tool for health and medical research. Singap Med J. 2008;49(3):256–60. quiz 61. 18363011

[42] Korstjens I, Moser A, Series. Practical guidance to qualitative research. Part 4: trustworthiness and publishing. Eur J Gen Pract. 2018;24(1):120–4. 29202616 10.1080/13814788.2017.1375092 PMC8816392

[43] Cristancho SM, Goldszmidt M, Lingard L, Watling C. Qualitative research essentials for medical education. Singap Med J. 2018;59(12):622–7. 10.11622/smedj.2018093 PMC6301871 30009321

[44] Ratelle JT, Bonnes SL, Wang AT, Mahapatra S, Schleck CD, Mandrekar JN, et al. Associations between teaching effectiveness and participant self-reflection in continuing medical education. Med Teach. 2017;39(7):697–703. 28301975 10.1080/0142159X.2017.1301655

[45] Ingram JR, Anderson EJ, Pugsley L. Difficulty giving feedback on underperformance undermines the educational value of multi-source feedback. Med Teach. 2013;35(10):838–46. 23808684 10.3109/0142159X.2013.804910

[46] Mather CA, McKay A, Allen P. Clinical supervisors’ perspectives on delivering work integrated learning: a survey study. Nurse Educ Today. 2015;35(4):625–31. 25618610 10.1016/j.nedt.2014.12.021

[47] Brown JM, Lowe K, Fillingham J, Murphy PN, Bamforth M, Shaw NJ. An investigation into the use of multi-source feedback (MSF) as a work-based assessment tool. Med Teach. 2014;36(11):997–1004. 24804910 10.3109/0142159X.2014.909920

[48] Khalife R, Gupta M, Gonsalves C, Park YS, Riddle J, Tekian A, et al. Patient involvement in assessment of postgraduate medical learners: A scoping review. Med Educ. 2022;56(6):602–13. 34981565 10.1111/medu.14726

[49] Øvrebø LJ, Dyrstad DN, Hansen BS. Assessment methods and tools to evaluate postgraduate critical care nursing students’ competence in clinical placement. An integrative review. Nurse Educ Pract. 2022;58:103258. 34847502 10.1016/j.nepr.2021.103258

[50] Araújo AAC, Godoy S, Maia N, Oliveira RM, Vedana KGG, Sousa ÁFL, et al. Positive and negative aspects of psychological stress in clinical education in nursing: A scoping review. Nurse Educ Today. 2023;126:105821. 37080012 10.1016/j.nedt.2023.105821

[51] Andersen T, Watkins K. The value of peer mentorship as an educational strategy in nursing. J Nurs Educ. 2018;57(4):217–24. 29614190 10.3928/01484834-20180322-05

