# Supplementary material for: Clinical education: nursing students’ experiences with multisource feedback using a digital assessment instrument in the emergency medical Service - a qualitative study
Source: BMC Med Educ. 2025 Mar 18;25:391. doi: 10.1186/s12909-025-06950-0 (PMC11916943; doi:10.1186/s12909-025-06950-0)
Supplement: Supplementary file 1 — Supplementary Material 1 [file 12909_2025_6950_MOESM1_ESM.docx]

Appendix 1: Codes, Categories and Themes

Introduction: This appendix presents the themes, categories, and codes as part of the original citations, all derived from reflexive thematic analysis. Following Braun and Clarke [2022, 2024], a non-positivist approach guided coding was used, ensuring coherence, researcher reflexivity, and meaning-based theme construction.

| **Themes** | **Categories** | **Sample quotations** |
| --- | --- | --- |
| Feedback from sources familiar with the LOs* | Self-reflection  Feedback from peers  Feedback from supervisor | *Then I recalled: What did I do regarding the LO? Did I do it well, and what could I have done differently.*  *I am so critical towards myself that it becomes straining.*  *I could support my peer using the peer-assessments.*  *I feel that if I don’t get feedback then I become lost* |
| Feedback from sources unfamiliar with the LOs | Feedback from patients  Feedback from other care professionals | *The patients are not familiar with what we students do.*  *I could not accept the feedback provided. I knew I did nothing wrong.*  *The handover needs to be comprehensible for the one who receives it and is supposed to pass it on in the organization wherefore, his or her feedback is super important.*  *Everyone is sitting inline, waiting to give their handover rapport and it doesn’t feel like the right time* |
| General perceptions of MSF** in the EMS*** | Context  Multisource feedback in the EMS | *Finding the right time for feedback is a challenge.*  *It would be easier, definitely. In a nursing department.*  *This instrument would be amazing to show at the assessment conference with all the different assessments. We could say, "Look, this is how the student has developed and progressed, with all these measurements and assessments."* |

*** LO- Learning Objectives ** MSF- Multisource Feedback *** EMS- Emergency medical service**

List of codes (Original and translated to English)

For the reader's convenience, the codes are presented in alphabetical order of original language and are presented in the original language first and then their translation to English. During data analysis the codes were examined for patterns and similarities. To reduce data complexity and uncover broader trends, related codes were grouped together. Finally, these grouped codes were organized into categories.

- AAI var en förutsättning - AAI was a prerequisite
- Annan struktur - Other structure
- Anonymitet - Anonymity
- Bekväm med återkoppling - Comfortable with feedback
- Beroendeställning - Dependency
- Beror på arbetsbelastning - Depends on workload
- Beskrivningar i stället för siffror - Descriptions instead of numbers
- Bias - Bias
- Borde skattat oftare - Should have assessed more often
- Bra - Good
- Bra med kamratåterkoppling - Good with peer feedback
- Bra med reflektion - Good with reflection
- Bra med studentåterkoppling - Good with student feedback
- Bra med veckosammanställning - Good with weekly summaries
- Bra tips - Good tips
- Bra återkoppling ifrån patienter - Good feedback from patients
- Bättre med anonym återkoppling - Better with anonymous feedback
- Bättre på avdelning - Better on the ward
- Diskussioner - Discussions
- Diskussioner gav mest - Discussions gave the most
- Dokumentation är bra - Documentation is good
- Effektivt - Effective
- En om dagen - One per day
- En/dag - One per day
- Endast bemötande kan bedömmas - Only behaviour can be assessed.
- Faciliterade för negativ återkoppling - Facilitated for negative feedback
- Faciliterar - Facilitates
- Fler perspektiv är positivt - More perspectives are positive
- Formulering - Formulation
- Forskningen påverkar negativt - Research negatively affects
- Få anhöriga - Few relatives
- Handledare ansvariga för återkoppling - Supervisors responsible for feedback
- Handledare bör ansvara för återkoppling - Supervisors should be responsible for feedback
- Handledare och självskattning bäst - Supervisors and self-assessment are best
- Handledarna ansvariga - The supervisors are responsible.
- Handledarna bör inhämta feedback - The supervisors should collect feedback.
- Hög belastning - High workload
- Ickeverbal återkoppling - Non-verbal feedback
- Individanpassa - Individualize
- Interaktion med mål - Interaction with goals
- Intrång i integreteten - Breach of integrity
- Jobbigt med negativ återkoppling - Difficult with negative feedback
- Jätteviktigt - Very important
- Kamratåterkoppling främjer diskussion - Peer feedback promotes discussion
- Lärorikt - Educational
- Lätt att göra - Easy to do
- Lättare på vårdavdelning - Easier on the ward
- Medicinskt tillstånd påverkar - Medical condition affects
- Mer konkret återkopling - More concrete feedback
- Mer reflektion - More reflection
- Minskad risk för misstag - Reduced risk of mistakes
- Mycket återkoppling - A lot of feedback
- Mål i rel till patient - Goals related to the patient.
- Möjlighet till reflektion - Opportunity for reflection
- Naturligare på vårdavdelning - More natural on the ward
- Negativ återkoppling - Negative feedback
- Negativt med en handledare - Negative with one supervisor
- Negativt med för mycket återkoppling - Negative with too much feedback
- Närvarande mål - Present goals
- Obekvämt - Uncomfortable
- Obekvämt med negativ återkoppling - Uncomfortable with negative feedback
- Obekvämt med patientåterkoppling - Uncomfortable with patient feedback
- Objektivt - Objective
- Olika handledare - Different supervisors
- Olika perspektiv - Different perspectives
- Olika perspektiv är viktigt - Different perspectives are important
- Ospecifik återkoppling - Unspecific feedback
- Ovana - Unfamiliarity
- Paddor är bra - Tablets are good
- Patientens tillstånd - Patient's condition
- Patienter förstå inte målen - Patients do not understand the goals
- Patienter förstår inte lärandemål - Patients do not understand learning objectives
- Patienter kan inte återkoppla - Patients cannot give feedback
- Patienterna ger bättre beskrivning - Patients provide better descriptions
- Perspektiv - Perspective
- Positivt med flera handledare - Positive with multiple supervisors
- Positivt med flera källor - Positive with multiple sources
- Positivt med perspektiv - Positive with perspectives
- Positivt med vårdkollegial återkoppling - Positive with feedback from healthcare colleagues
- Positivt med återkoppling - Positive with feedback
- Reflektion direkt efter vårdmöte - Reflection immediately after patient meeting
- Reflektion ger dialog - Reflection creates dialogue
- Relation viktigt för återkoppling - Relationship is important for feedback
- Relation är viktig för återkoppling - Relationship is important for feedback
- Riktade lärandemål till patienter - Directed learning goals to patients
- Roligt med återkoppling ifrån patienter - Fun with feedback from patients
- Rättvist - Fair
- Sammanställning - Summary
- Sammanställning positiv - Positive summary
- Sammanställningen säger inget - The summary says nothing
- Sammanställning - Compilation
- Sedd - Seen
- Selektionsbias - Selection bias
- Sett andras utveckling - Seen others' development
- Siffror är ospecifikt - Numbers are unspecific
- Sinnesstämning påverkar - Mood affects
- Sjukdomstillstånd påverkar - Medical condition affects
- Självkritisk - Self-critical
- Självskattning ger reflektion - Self-assessment provides reflection
- Skapar diskussion - Creates discussion
- Språkbarriär - Language barrier
- Stressigt att vara själv - Stressful to be alone
- Struktur - Structure
- Struktur är viktigt - Structure is important
- Student och handledare ger bra återkoppling - Student and supervisor provide good feedback
- Stöd - Support
- Stöd vid utmaningar - Support during challenges
- Stötta varandra - Support each other
- subjektiv återkoppling ifrån patient - Subjective feedback from patient
- Svårhanterligt - Difficult to handle
- Svårt - Difficult
- Svårt att koppla ihop bedömningar - Difficult to connect evaluations
- Svårt att vara kritisk - Difficult to be critical
- Svårt med tajming - Difficult with timing
- Svårt i kontexten - Difficult in context
- Svårt med samarbete - Difficult with collaboration
- Svårt med sjuka patienter - Difficult with sick patients
- Svårt med tajming - Difficult with timing
- Svårt på nätter - Difficult at nights
- Svåra lärandemål - Difficult learning goals
- System tydliggörs av flera perspektiv - System clarified by multiple perspectives
- Tajming - Timing
- Tajming viktigt för återkoppling - Timing is important for feedback
- Tid till återkoppling - Time for feedback
- Tidsbrist - Lack of time
- Tydligare på vårdavdelning - Clearer on the ward
- Tydligt - Clear
- Tydligt med veckorapporter - Clear with weekly reports
- Vilse utan - Lost without
- Viktigt - Important
- Vägledning - Guidance
- Återkoppling ger reflektion - Feedback provides reflection
- Återkoppling driver diskussion - Feedback drives discussion
- Återkoppling ger reflektion - Feedback provides reflection
- Önskar mer feedback - Wish for more feedback
