# Supplementary material for: Clinical education: nursing students’ experiences with multisource feedback using a digital assessment instrument in the emergency medical Service - a qualitative study
Source: BMC Med Educ. 2025 Mar 18;25:391. doi: 10.1186/s12909-025-06950-0 (PMC11916943; doi:10.1186/s12909-025-06950-0)
Supplement: Supplementary file 2 — Supplementary Material 2 [file 12909_2025_6950_MOESM2_ESM.docx]

Consolidated criteria for reporting qualitative studies (COREQ) 32 item checklist

| **No.** | **Item** | **Guide question** | **Answer** |
| --- | --- | --- | --- |
| **Domain 1: Research team and reflexivity** |  |  |  |
| Personal Characteristics |  |  |  |
| 1 | Interviewer/facilitator | Which author/s conducted the interview or focus group? | Main author |
| 2 | Credentials | What were the researcher's credentials? E.g. PhD, MD | 2 PhD  2 Professors |
| 3 | Occupation | \| What was their occupation at the time of the study? \| \| --- \| | Clinicians, head of research and university teachers |
| 4 | Gender | Was the researcher male or female? | Head researcher is male. Research team is 2 male and 2 female |
| 5 | Experience and training | What experience or training did the researcher have? | All researchers were nurses |
| Relationship with participants |  |  |  |
| 6 | Relationship established | Was a relationship established prior to study commencement? | A relationship was not established before the study. |
| 7 | Participant knowledge of the interviewer | What did the participants know about the researcher? e.g. personal goals, reasons for doing the research | Participants had no prior knowledge before the study, but it is likely that knowledge was gained throughout the study. The main author is well known in the given context. |
| 8 | Interviewer characteristics | What characteristics were reported about the interviewer/facilitator? e.g. Bias, assumptions, reasons, and interests in the research topic | The participants was informed about the researchers prior experience as educational supervisors and clinical experience |
| **Domain 2: study design** |  |  |  |
| Theoretical framework |  |  |  |
| 9 | Methodological orientation and Theory | What methodological orientation was stated to underpin the study? e.g. grounded theory, discourse analysis, ethnography, phenomenology, content analysis | A form of content analysis was used. Reflexive, thematic analysis by Brown and Clarke |
| Participant selection |  |  |  |
| 10 | Sampling | How were participants selected? e.g. purposive, convenience, consecutive, snowball | A convenience sample was used |
| 11 | Method of approach | How were participants approached? e.g. face-to-face, telephone, mail, email | The students were initially approached in a joint meeting for all students before the clinical education started |
| 12 | Sample size | How many participants were in the study? | 31 |
| 13 | Non-participation | How many people refused to participate or dropped out? Reasons? | 1 |
| Setting |  |  |  |
| 14 | Setting of data collection | Where was the data collected? e.g. home, clinic, workplace | The data was collected in one of the main ambulance stations in Stockholm |
| 15 | Presence of non-participants | Was anyone else present besides the participants and researchers? | One supervisor partook as an assistant to the researcher during the focus groups |
| 16 | Description of sample | What are the important characteristics of the sample? e.g. demographic data, date | It was centrally located in Stockholm areas |
| Data collection |  |  |  |
| 17 | Interview guide | Were questions, prompts, guides provided by the authors? Was it pilot tested? | Yes. An interview guide was used. It was not piloted |
| 18 | Repeat interviews | Were repeat interviews carried out? If yes, how many? | Four focus groups were conducted |
| 19 | Audio/visual recording | Did the research use audio or visual recording to collect the data? | Audio recordings were used |
| 20 | Field notes | Were field notes made during and/or after the interview or focus group? | Notes were taken by the assistant supervisor |
| 21 | Duration | What was the duration of the interviews or focus group? | 45-70 minutes |
| 22 | Data saturation | Was data saturation discussed? | Data saturation was discussed |
| 23 | Transcripts returned | Were transcripts returned to participants for comment and/or correction? | Transcripts were not returned to the participants |
| **Domain 3: analysis and findings** |  |  |  |
| Data analysis |  |  |  |
| 24 | Number of data coders | How many data coders coded the data? | 1 main coder and 3 reference coders |
| 25 | Description of the coding tree | Did authors provide a description of the coding tree? | The coding tree was discussed in the research group |
| 26 | Derivation of themes | Were themes identified in advance or derived from the data? | Themes derived from the data |
| 27 | Software | What software, if applicable, was used to manage the data? | Microsoft Exel was used |
| 28 | Participant checking | Did participants provide feedback on the findings? | No |
| Reporting |  |  |  |
| 29 | Quotations presented | Were participant quotations presented to illustrate the themes / findings? Was each quotation identified? e.g. participant number | Quatations were used and linked back to the focus group but not on an individual level. |
| 30 | Data and findings consistent | Was there consistency between the data presented and the findings? | There was consistency between the data and findings |
| 31 | Clarity of major themes | Were major themes clearly presented in the findings? | Yes |
| 32 | Clarity of minor themes | Is there a description of diverse cases or discussion of minor themes? | The participants sometimes disagreed and therefore there were conflicted statements in the findings. However, on a general level there were agreement. |
