# Supplementary material for: Clinical education: nursing students’ experiences with multisource feedback using a digital assessment instrument in the emergency medical Service - a qualitative study
Source: BMC Med Educ. 2025 Mar 18;25:391. doi: 10.1186/s12909-025-06950-0 (PMC11916943; doi:10.1186/s12909-025-06950-0)
Supplement: Supplementary file 3 — Supplementary Material 3 [file 12909_2025_6950_MOESM3_ESM.docx]

Appendix 2 - Ambulance Assessment Instrument

Introduction:

This appendix presents the Ambulance Assessment Instrument (AAI), an instrument designed to systematically evaluate students during clinical education in emergency medical services. It ensures structured assessments, providing feedback to enhance educational quality. All learning objectives were designed to be used by supervisors, peers and the students themselves for self-reflection. Learning objective 1,2,3 and 4 was designed for patients. Learning objectives 5 and 6 was designed for other healthcare professionals. The instrument was validated by Nilsson et al [2024]

Learning objective 1 (For patients & next of kin)

To what extent did the student treat the patient with respect?

| 1 | 2 | 3 | 4 | 5 | 6 | 7 |
| --- | --- | --- | --- | --- | --- | --- |
| Not at all |  | | | | | To a very high degree |

Learning objective 2 (For patients & next of kin)

To what extent did the student provide the patient with an opportunity to describe their situation?

| 1 | 2 | 3 | 4 | 5 | 6 | 7 |
| --- | --- | --- | --- | --- | --- | --- |
| Not at all |  | | | | | To a very high degree |

Learning objective 3 (For patients & next of kin)

To what extent did the student create a safe environment for the patient?

| 1 | 2 | 3 | 4 | 5 | 6 | 7 |
| --- | --- | --- | --- | --- | --- | --- |
| Not at all |  | | | | | To a very high degree |

Learning objective 4 (For patients & next of kin)

To what extent did the student inform the patient of the examinations that would be performed?

| 1 | 2 | 3 | 4 | 5 | 6 | 7 |
| --- | --- | --- | --- | --- | --- | --- |
| Not at all |  | | | | | To a very high degree |

Learning objective 5 (For other healthcare professional)

To what extend did the student convey information related to the patient’s needs.

| 1 | 2 | 3 | 4 | 5 | 6 | 7 |
| --- | --- | --- | --- | --- | --- | --- |
| Not at all |  | | | | | To a very high degree |

Learning objective 6 (For other healthcare professional)

To what extent did the student perform a provide a structured oral report?

| 1 | 2 | 3 | 4 | 5 | 6 | 7 |
| --- | --- | --- | --- | --- | --- | --- |
| Not at all |  | | | | | To a very high degree |
|  |  | | | | |  |

Learning objective 7

To what extent was the student familiar with the equipment used in the caring situation?

| 1 | 2 | 3 | 4 | 5 | 6 | 7 |
| --- | --- | --- | --- | --- | --- | --- |
| Not at all |  | | | | | To a very high degree |

Learning objective 8

To what extent did the student display a good ability to observe and execute risk assessments of behaviour, signs and symptoms of acute failure in health?

| 1 | 2 | 3 | 4 | 5 | 6 | 7 |
| --- | --- | --- | --- | --- | --- | --- |
| Not at all |  | | | | | To a very high degree |

Learning objective 9

To what extent did the student use the technology in collaboration with the patient?

| 1 | 2 | 3 | 4 | 5 | 6 | 7 |
| --- | --- | --- | --- | --- | --- | --- |
| Not at all |  | | | | | To a very high degree |

Learning objective 10

To what extent did the student plan and prioritize between caring measures from the patient’s perspective?

| 1 | 2 | 3 | 4 | 5 | 6 | 7 |
| --- | --- | --- | --- | --- | --- | --- |
| Not at all |  | | | | | To a very high degree |

Learning objective 11

To what extent did the student analyse the consequences of prioritization made? (For other healthcare professional)

| 1 | 2 | 3 | 4 | 5 | 6 | 7 |
| --- | --- | --- | --- | --- | --- | --- |
| Not at all |  | | | | | To a very high degree |

Learning objective 12

To what extent did the student treat the patient according to the principle of equal treatment?

| 1 | 2 | 3 | 4 | 5 | 6 | 7 |
| --- | --- | --- | --- | --- | --- | --- |
| Not at all |  | | | | | To a very high degree |

Learning objective 13

To what extent did the student perform the caring procedures in accordance with aseptic and hygienic principles?

| 1 | 2 | 3 | 4 | 5 | 6 | 7 |
| --- | --- | --- | --- | --- | --- | --- |
| Not at all |  | | | | | To a very high degree |
